# Recto Sigmoid Sub-Mucosal Bleeding Due to Warfarin over Anticoagulation Presenting as Intestinal Obstruction

**DOI:** 10.4314/ejhs.v35i1.9

**Published:** 2025-01

**Authors:** Fithanegest Teferra Gebretekle, Minyahil Zeleke Tesfaye, Tibebu Tesfaye

**Affiliations:** 1 Department of Radiology, St. Paul's Hospital Millennium Medical College, Addis Ababa, Ethiopia; 2 Department of Internal Medicine, Girum General Hospital, Addis Ababa, Ethiopia; 3 Department of Surgery, Girum General Hospital, Addis Ababa, Ethiopia

**Keywords:** Warfarin, Submucosal, Obstruction, Bleeding, Surgery/Laparotomy, hematoma, over anti-coagulation and INR

## Abstract

Patients on anticoagulation therapy, particularly those on warfarin, are at risk of gastrointestinal bleeding, gum bleeding, hematuria, and ecchymosis. However, it is rare for such patients to present with intramural or submucosal bleeding leading to intestinal obstructive symptoms. Sub-mucosal intestinal bleeding due to prolonged anticoagulant use is uncommon. Literature suggests that the duodenum and small intestine are common locations for anticoagulant-induced hematomas, occurring in approximately 1 case per 2,500 anticoagulated patients per year. However, intramural colonic hematomas are rarely reported. Spontaneous anticoagulant induced hematomas may develop as early as 10 days after starting therapy. We report the case of a 63-year-old female who presented with recto-sigmoid sub-mucosal bleeding causing obstructive symptoms. The patient was managed surgically with laparotomy, resulting in significant improvement, and was scheduled for follow-up to evaluate the feasibility of reintroducing anticoagulation therapy.

## Introduction

Warfarin is commonly used for long-term thromboprophylaxis in conditions such as pulmonary embolism, deep vein thrombosis, atrial fibrillation, and in patients with prosthetic heart valves (1-3). The target INR range is 2–3. However, anticoagulation therapy carries a risk of bleeding. In addition to the more common complications, such as gum bleeding, hematuria, hematemesis, and rectal bleeding, rare and severe cases of sub-mucosal or intramural intestinal hematomas, as well as intra- and retroperitoneal hemorrhages, have been reported (1-3, 7). Sub-mucosal hematomas of the large bowel are particularly rare but can be fatal (1, 5). For example, rectal submucosal bleeding can lead to shock and compression symptoms, which pose significant risks. Management may involve either conservative treatment or surgical exploration to control bleeding or decompress the affected area (1-2). This case report describes a patient with recto-sigmoid sub-mucosal bleeding due to warfarin toxicity, resulting in intestinal obstruction.

## Consent

Written informed consent was obtained from the patient for publication of this case report, including the use of accompanying images and investigation results. All investigations were conducted as part of her standard clinical care, with full approval and understanding from the patient. Patient confidentiality has been maintained, and all personal data has been anonymized. Formal institutional ethical approval was also obtained following institutional guidelines.

## Case Report

A 63-year-old divorced female presented on July 7, 2022, with sudden onset crampy abdominal pain, abdominal distension, vomiting, and brief episodes of fainting and dizziness. The abdominal pain was generalized but more localized to the left iliac fossa. She had been diagnosed with post-COVID deep vein thrombosis (DVT) three months prior and had been on warfarin (5 mg PO daily). Her last INR, taken 30 days before her current presentation, was 2.83.

**Figure 1:**
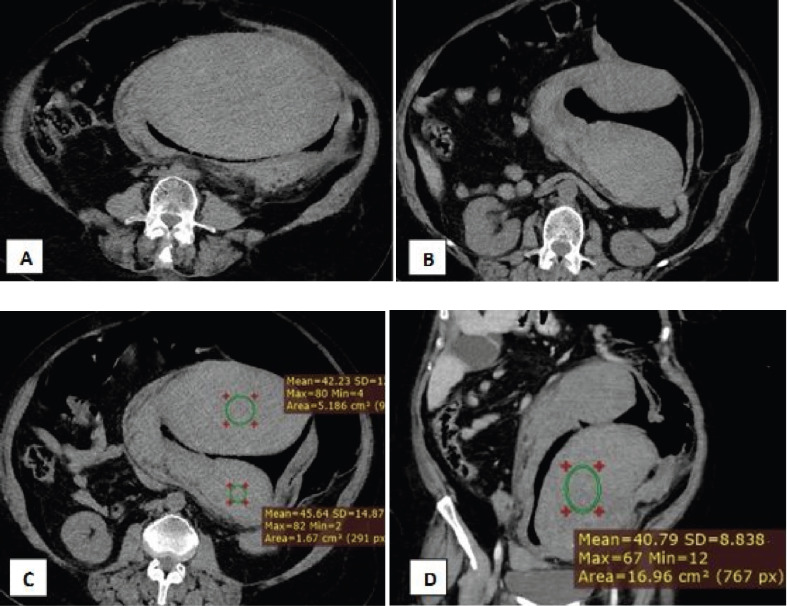
Axial [A-C] and coronal [D] Computed Tomography of abdomen showing homogeneously hyperattenuating submucosal mass involving the sigmoid colon [attenuation 40-45 Hu] which extends along the entire length of the sigmoid colon suggested a hematoma, led to the decision for surgical intervention

On examination, her abdomen was distended with hypoactive bowel sounds, and she had a large, well-defined palpable mass in the left lower quadrant. On presentation, she was in shock, requiring three bags of normal saline, and was transferred to the ICU. Laboratory results showed a hemoglobin of 9.9 g/dl, WBC count of 23.3k, and platelets of 186k. Her INR was 4.27, and PT was 49 seconds. A CT scan revealed a large hyperdense mass along the entire sigmoid colon, suggesting a submucosal hematoma, with a density of 44 HU, causing marked luminal narrowing, and evidence of cholelithiasis (Figure 1).

The patient was kept NPO and received two units of whole blood and three units of fresh frozen plasma (FFP) to stabilize her condition. Intravenous Vitamin K was administered, anticoagulation therapy was discontinued, and low-dose noradrenaline was initiated for hemodynamic support. Initially, a conservative approach was adopted, anticipating spontaneous recovery and concerns about surgical bleeding. However, her abdominal distension worsened, and she was unable to pass stool or gas. Her INR dropped to 2.03, but her hemoglobin continued to decrease, reaching 8.2 g/dL. Additional transfusions of two units of packed red blood cells and four units of FFP were given.

**Figure 2:**
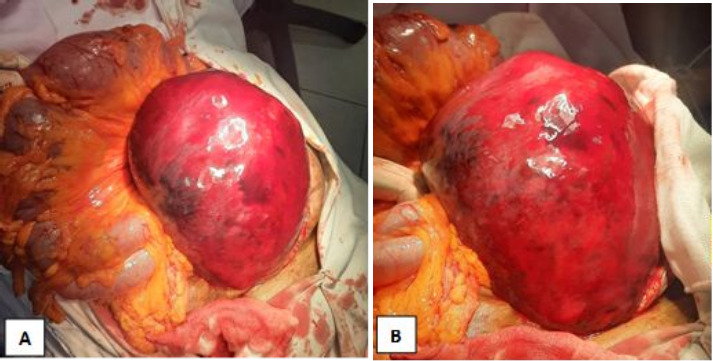
[A & B], an intraoperative image showing a large and shiny sigmoid mass lesion suggesting hematoma extending to the rectum with mildly distended adjacent loops of bowel

With her condition worsening, the surgical team opted for emergency surgery. An exploratory laparotomy revealed a significant intramural hematoma in the sigmoid colon, extending to the proximal rectum. The patient underwent en bloc resection with Hartmann's colostomy (Figure 2).

Over the following week, the patient's condition improved, with her INR decreasing to 1.62 and hemoglobin rising to 9.8 g/dL. Her abdominal pain and distention resolved, and there were no further bleeding episodes. Biopsy results showed colonic tissue with sub-mucosal and sub-serosal hemorrhage, associated with neutrophilic inflammatory cell infiltrates, but no malignancy. One week after discharge, the patient was stable and resumed normal activities. A follow-up colon anastomosis surgery was planned.

## Discussion

Warfarin, used for anticoagulation in thromboembolic conditions, carries a risk of bleeding, especially if the INR exceeds the therapeutic range of 2-3. Elevated INR can lead to spontaneous gastrointestinal bleeding, intracerebral hemorrhage, hematuria, hemarthrosis, and, less commonly, intraperitoneal or retroperitoneal hemorrhage. Contributing factors to elevated INR include higher warfarin doses, old age, poor INR monitoring, and drug-drug interactions, such as those with NSAIDs, Clopidogrel, and cytochrome P450 enzyme inhibitors (3, 7).

Patients on warfarin may present with shock or intestinal obstruction due to bleeding in the submucosal or intramural layers. These hematomas can be managed conservatively by withholding anticoagulants, administering intravenous Vitamin K (to correct INR within 24 hours), and transfusing fresh frozen plasma (15 ml/kg) and whole blood if necessary. Surgical intervention may be required if conservative measures fail or if signs of bowel necrosis, peritonitis, or persistent obstruction are present (1,3, 7-8).

In this case, the patient was initially managed conservatively, but her condition deteriorated due to ongoing bleeding, necessitating surgery. Surgical management of anticoagulant-induced hematomas is challenging, particularly due to the risk of hemorrhage during the procedure. Fortunately, there were no significant complications during surgery.

Early recognition and appropriate management of anticoagulant-induced bleeding are crucial. Imaging studies, such as CT scans, can help identify the source of bleeding and guide management, avoiding unnecessary surgery (2, 4). This case emphasizes the importance of regular INR monitoring and educating patients on the risks associated with anticoagulant therapy. Identifying over-anticoagulation early can prevent severe complications like intestinal obstruction. Additionally, drug-drug interactions should be considered when prescribing other medications to patients on warfarin.

Reintroducing anticoagulant therapy after resolving a hematoma should be done cautiously, with careful risk-benefit evaluation, as recurrent hematomas have been reported in up to 60% of cases after restarting therapy (2, 7-8).
